# Clinical profile and analysis of adherence to self-care in patients with type 2 diabetes: a cross-sectional study, Goiânia, Goiás, Brazil, 2024

**DOI:** 10.1590/S2237-96222026v35e20250161.en

**Published:** 2026-05-02

**Authors:** Isabela Alves Cunha, Ana Caroline de Oliveira Lima, Lucas Raphael Bento Silva, Jordana Campos Martins de Oliveira, Geovana Cristina Batista Pacheco

**Affiliations:** 1 Hospital Estadual Alberto Rassi Comissão de Residência Multiprofissional Goiânia GO Brazil Hospital Estadual Alberto Rassi, Comissão de Residência Multiprofissional, Goiânia, GO, Brazil; 2 Centro Universitário Araguaia Goiânia Goiânia GO Brazil Centro Universitário Araguaia, Goiânia, GO, Brazil

**Keywords:** Diabetes Complications, Self-Management, Endocrinology, Noncommunicable Diseases, Cross-Sectional Studies, Complicaciones de la Diabetes, Automanejo, Endocrinología, Enfermedades no Transmisibles, Estudios transversales

## Abstract

**Objective:**

To characterize the clinical profile of patients with type 2 diabetes and analyze their adherence to self-care.

**Methods:**

A cross-sectional epidemiological study was conducted at a state-level center specializing in diabetes care in Goiânia, Goiás state, Brazil, between August and November 2024. The study involved administration of a sociodemographic questionnaire, investigation of health history, anthropometric assessment and administration of the Diabetes Self-Care Activities Questionnaire (DSCQ). The data are presented as absolute and relative frequencies, means and standard deviations. Associations were analyzed using multiple linear regression models, with a 95% confidence interval (95%CI) and a 5% significance level.

**Results:**

The sample of 199 participants had a mean age of 64.9 (9.3) years, predominance of females (66.30%), more than 10 years since diagnosis (67.30%), and more than 80% were overweight or obese. The majority were sedentary (84.40%), non-smokers (94.50%) and non-drinkers (77.90%). Regarding diabetes complications, 45.60% reported having only one, with neuropathy being the most frequently cited (64.32%), followed by retinopathy (25.12%) and nephropathy (13.56%). The DSQC revealed low adherence to self-management, especially regarding physical activity (88.90%), following professional dietary guidelines (63.80%), avoiding fatty foods (62.30%), monitoring blood glucose (63.80%) and examining feet (64.30%). There was unsatisfactory adherence to most DSQC items.

**Conclusion:**

These are individuals in their sixth decade of life, overweight or obese, diagnosed for more than 10 years and with low adherence to self-care.

## Resumen

**Objetivo:**

Caracterizar el perfil clínico de pacientes con diabetes tipo 2 y analizar su adherencia al autocuidado.

**Métodos:**

Se realizó un estudio epidemiológico transversal en un centro estatal especializado en diabetes en Goiânia, estado de Goiás, Brasil, entre agosto y noviembre de 2024. Se aplicó un cuestionario sociodemográfico, una investigación de antecedentes de salud, una evaluación antropométrica y el Cuestionario de Actividades de Autocuidado de la Diabetes (CAD). Los datos se presentan como frecuencias absolutas y relativas, media y desviación estándar. Las asociaciones se analizaron mediante modelos de regresión lineal múltiple, con un intervalo de confianza del 95% (IC95%) y un nivel de significancia del 5%.

**Resultados:**

La muestra de 199 participantes tenía una edad media de 64,9 (9,3) años, un predominio de mujeres (66,30%), más de 10 años desde el diagnóstico (67,30%) y más del 80% tenía sobrepeso u obesidad. La mayoría eran sedentarios (84,40%), no fumadores (94,50%) y no bebedores (77,90%). Con respecto a las complicaciones de la diabetes, el 45,60% reportó tener solo una, siendo la neuropatía la más frecuentemente citada (64,32%), seguida de la retinopatía (25,12%) y la nefropatía (13,56%). El CAD reveló baja adherencia al autocuidado, especialmente con respecto a la actividad física (88,90%), seguir las guías dietéticas profesionales (63,80%), evitar alimentos grasos (62,30%), monitorear la glucosa en sangre (63,80%) y examinar los pies (64,30%). La mayoría de los ítems del CAD mostraron una adherencia insatisfactoria.

**Conclusión:**

Se trata de personas en la sexta década de la vida, con sobrepeso u obesidad, con más de 10 años de diagnóstico y baja adherencia al autocuidado.

**Ethical aspects**

This research respected ethical principles, having obtained the following approval data:

Research ethics committee: Hospital Estadual Alberto Rassi

Opinion number: 6,030,934

Approval date: 17/4/2023

Certificate of submission for ethical appraisal: 67329423.4.0000.0035

Informed consent form: Obtained from all participants prior to data collection.

## Introduction

Diabetes is a chronic metabolic illness characterized by accumulation of serum glucose, influenced by external factors and clinical conditions [1]. Its etiology is composed of non-modifiable risk factors, such as age, sex and heredity, and modifiable factors, such as lifestyle habits [2]. Diabetes is classified into two types: type 1, which has a strong genetic predisposition and requires continuous treatment with insulin [3]; and type 2, whereby, although it also has a genetic component, controlling it is related to lifestyle changes [4].

Occurrence of type 2 diabetes increases with age and has higher incidence between 55 and 59 years of age, affecting men at younger ages [5]. In 2021, Brazil was the country with the sixth highest number of people with the disease and, according to estimates, this number will increase by 47% in 20 years [6].

Self-care routines for type 2 diabetes should include management of medication and lifestyle habits, such as proper nutrition, physical exercise, water intake, cessation of alcohol and/or tobacco use, and monitoring foot health to prevent chronic complications of the disease [7]. However, these individuals often have inadequate habits and lower adherence to non-pharmacological treatments [8]. Poor management of type 2 diabetes influences the appearance of new conditions [9], and individuals may present, for example, elevated body mass index (BMI), lipid deposition and osteomyoarticular dysfunction [8].

In this context, self-care assessment tools are important for mapping the main factors detrimental to the health and quality of life of people with type 2 diabetes [10]. Using questionnaires that analyze the level of adherence to treatment in these patients enables identification of problems and the need for guidance involving a self-care plan associated with clinical management [10]. To this end, the Diabetes Self-Care Activities Questionnaire (DSCQ) is a highly reliable instrument that assesses the level of adherence to self-care by analyzing the frequency of doing certain activities on a weekly basis [11].

In view of this, there is a need to investigate adherence of individuals with type 2 diabetes to their management activities in order to identify aspects that influence it. As such, this study aimed to characterize the clinical profile of patients with type 2 diabetes and analyze their adherence to self-care.

## Methods

### Design

This is a cross-sectional epidemiological study.

### Setting

This study was conducted at a state referral center for the care of people with diabetes, located in Goiânia, capital of the state of Goiás, from August to November 2024. Data from the State Health Department indicate that, in Goiás, self-reported prevalence of diabetes among adults is 6.4% [12]. In addition to prevalence, the rate of hospitalizations attributed to diabetes reached 57.44%, with the highest costs associated with elderly people with chronic complications of the disease [13]. These complications, frequently resulting from absence or inadequacy of self-care, constitute the main focus of this investigation, given their significant impact on morbidity and mortality and health costs [8,9].

### Participants

Individuals over 18 years of age, of both sexes, diagnosed with type 2 diabetes, with a minimum of three months of follow-up at the health institution, and who signed the informed consent record were included in the study. Exclusion criteria were individuals unable to answer the anamnesis due to cognitive and/or biological issues or who chose to withdraw their consent to participate in the research.

The researchers presented the informed consent record to the volunteers and informed them about the objectives and procedures they would undergo, as well as all the risks and benefits.

### Variables

The dependent variables were self-care behaviors measured by the DSCQ, encompassing the domains of “overall diet”, “specific diet”, “physical activity”, “blood glucose monitoring”, “foot care”, “medication use” and “smoking”.

The independent variables were as follows:

Sociodemographic data:

- sex (male; female);
- age group (in years: 18-29; 30-39; 40-49; 50-59; 60-69; 70-79; 80-85);
- monthly income (less than one minimum wage; between one and two minimum wages; more than two minimum wages); and
- schooling (no schooling; incomplete elementary; complete elementary; incomplete high school; complete high school; incomplete higher education; complete higher education).

Clinical data:

- weight (in kilograms – kg);
- height (in meters – m);
- BMI (in kilograms per square meter – kg/m^2^; classified as: healthy weight, overweight, obesity);
- tobacco smoking (yes; no);
- alcohol use (yes; no);
- time since diagnosis (<10 years since diagnosis; ≥10 years since diagnosis);
- doing physical exercise (active; sedentary);
- medication therapy (tablets; insulin therapy; both);
- number of complications (none; only one; two associated; three associated);
- amputation (yes; no);
- history of lesions (yes; no); and
- acute lesions (yes; no).

### Data sources and measurement

The data were collected by means of interviews and questionnaires administered by trained professionals experienced in the treatment of type 2 diabetes. The language was adapted to the target audience to ensure understanding of the topics covered and reliability of the responses. Cognitive deficits were determined based on the professionals’ perception during the interview, backed by analysis of medical records.

A sociodemographic questionnaire compiled by the authors themselves was used to collect sociodemographic data and health history, with questions regarding age, sex, monthly income, education level, weight, height, BMI, time since diagnosis of type 2 diabetes, diabetic complications, medication therapy, smoking, alcohol consumption, physical exercise, history of foot lesions and amputation.

The DSCQ was administered for the purpose of assessing self-care. It is a revised and standardized questionnaire with scores that assess adherence of individuals with type 2 diabetes to self-care as part of treatment of the disease [14]. The translated, adapted and validated version of the DSCQ for use in Portuguese was administered [15]. The DSCQ is composed of 15 items that analyze five aspects of diabetes treatment, grouped into seven dimensions of self-care: i) overall diet; ii) specific diet; iii) physical activity; iv) blood glucose monitoring; v) foot care; vi) medication use; and vii) smoking [16].

For each item, the patient is asked about the number of days per week they do a particular self-care activity [16]. The alternatives range from 0 to 7 days, with “0 days” considered the least expected response, indicating lower adherence to self-care, and “7 days” the most expected, indicating better self-management of the disease [17]. However, in the last two items of the “specific diet” dimension, the values ​​are reversed, whereby the patient is asked about consumption of foods high in fat and sugar [17]. For these items, the response “0 days” is the most expected, and the response “7 days” is the least expected [17].

Behavior is classified as desirable when the average number of days for each item is greater than 4 or, in the case of items 2.2 and 2.3 of the “specific diet” dimension, when the average number of days is ≤4 [18]. The “overall diet”, “physical activity” and “blood glucose monitoring” dimensions, due to having good correlations between their items, were assessed according to total mean number of days between them [15]. The items of the other dimensions were assessed individually and presented as frequencies, due to the weak correlation between them [15].

### Bias

Some data were based on self-reported information, which is subject to both information bias and recall bias, meaning participants might forget relevant aspects of their health history.

### Study size

This is a non-probabilistic convenience sample. The sample was based on the work schedules of health professionals at a specialized state center, including doctors, nutritionists, podiatrists, nurses and physiotherapists. Patients invited to participate in the research were approached on Wednesdays and Fridays in the morning while waiting for their appointments in the waiting room. After being approached, they were directed to a private room with good lighting and air conditioning to ensure patient privacy.

Considering an average of 15 patients per period, the data collection period lasted four months, from August to November 2024. This strategy aimed to align the selection with the institution’s routine. The selection stages are shown in Figure 1.

**Figure 1:**
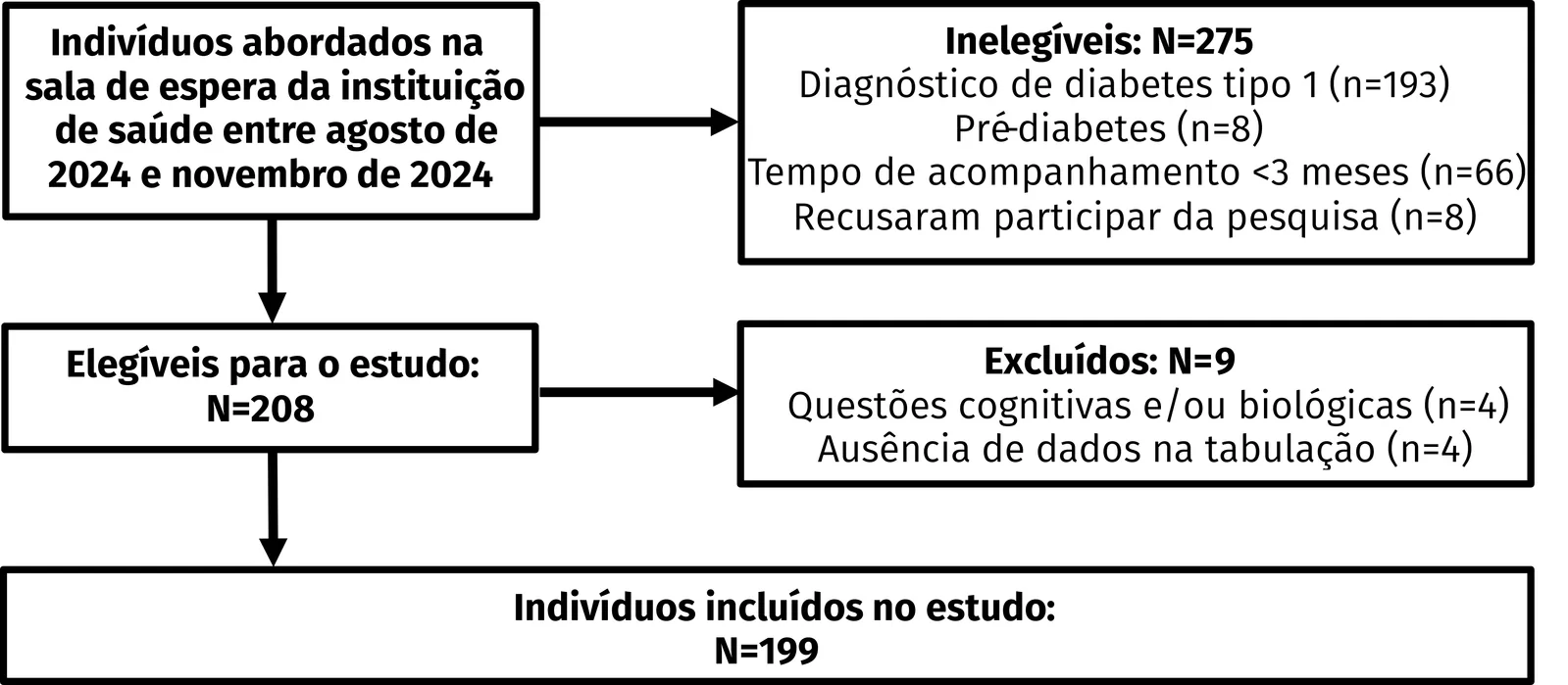
Stages for study participant eligibility and selection. Goiânia, Goiás, 2024 (n= 199)

### Statistical methods

In descriptive terms, the continuous variables were expressed as mean and standard deviation, while the categorical variables were presented as absolute and relative frequencies.

Multiple linear regression models were used to investigate associations between sociodemographic and clinical variables and self-care scores (assessed using the DSCQ), taking the means of the DSCQ dimensions as continuous outcomes. The model was adjusted for variables such as sex, age, BMI, time since diagnosis, education level and income. The assumptions of the linear regression models were checked, including normality of residuals, homoscedasticity and absence of multicollinearity. The results were expressed by the estimated beta (β) coefficients, with respective 95% confidence intervals (95%CI). The data were analyzed using the Statistical Package for the Social Sciences (SPSS) software, version 25.0, taking a fixed 5% significance level.

Statistical significance was interpreted with caution, also considering the magnitude of the effects and their clinical relevance, in accordance with current methodological recommendations in the field of public health [19].

## Results

The study was conducted with a sample of 199 patients. Mean age was 64.9 (9.3) years, with 43.7% being in the sixth decade of life and with females predominating (66.3%). Most individuals reported earning more than one minimum wage per month (92.0%). Only 1.5% of individuals had completed a university degree and 12.1% had never attended educational institutions [Table 1].

**Table 1:** Sociodemographic characterization of the sample shown by absolute and relative frequency. Goiânia, Goiás, 2024 (n=199)

Sample characteristics	Values n (%)
Sex	
Male	67 (33.7)
Female	132 (66.3)
**Age group **(years)	
18-29	0 (0)
30-39	2 (1)
40-49	9 (4.5)
50-59	43 (21.6)
60-69	87 (43.7)
70-79	48 (24.1)
80-85	10 (5.1)
**Monthly income **(**minimum wages**)	
<1	16 (8)
1-2	118 (59.3)
>2	65 (32.7)
Schooling	
No schooling	24 (12.1)
Incomplete elementary	92 (46.2)
Complete elementary	12 (6)
Incomplete high school	16 (8)
Complete high school	49 (24.6)
Incomplete higher education	3 (1.5)
Complete higher education	3 (1.5)

The average weight of the sample was 77.1 (16.1) kg and average height was 1.61 (0.09) m, resulting in an average BMI of 29.6 (5.7) kg/m^2^, with 41.2% classified as overweight and 40.2% as obese. The majority of the sample consisted of non-smokers (94.5%), non-drinkers (77.9%) and sedentary individuals (84.4%). Most had been diagnosed for more than 10 years (67.3%), used only tablets as medication therapy (51.3%) and presented only one complication related to type 2 diabetes (45.6%). Regarding complications, neuropathy was the most frequently cited (64.32%), followed by retinopathy (25.12%) and nephropathy (13.56%). Other data characterizing the clinical profile of the sample are presented in Table 2.

**Table 2:** Characterization of the clinical profile of patients with type 2 diabetes shown by absolute and relative frequency. Goiânia, Goiás, 2024 (n=199)

Variable	Values n (%)
**BMIa classification **	
Healthy weight	37 (18.6)
Overweight	82 (41.2)
Obesity	80 (40.2)
**Tobacco smoking **	
Smoker	11 (5.5)
Non-smoker	188 (94.5)
**Alcohol use **	
Alcohol user	44 (22.1)
Non-alcohol user	155 (77.9)
**Time since diagnosis **(years)	
<10	65 (32.7)
≥10	134 (67.3)
**Physical exercise**	
Active	31 (15.6)
Sedentary	168 (84.4)
**Medication therapy**	
Tablets	102 (51.3)
Insulin therapy	12 (6)
Both	85 (42.7)
**Hospitalization in the last two years due to diabetes decompensation**	
Yes	33 (16.6)
No	166 (83.4)
**Number of diabetes complications**	
None	56 (28.4)
Only one	91 (45.6)
Two associated	42 (21)
Three associated	10 (5)
**Presence of amputation **	
Amputation present	17 (8.5)
Amputation not present	182 (91.5)
**History of foot lesions **	
Yes	64 (32.2)
No	135 (67.8)
**Current presence of foot lesions**	
Yes	21 (11)
No	178 (89)

^a^Body Mass Index.

The DSCQ activities regarding physical exercise (items 3.1 and 3.2) showed the poorest adherence. Other activities with low adherence were: i) item 1.2 – “followed professional dietary guidance”, with 63.8% reporting less than 4 days per week; ii) item 2.2 – “ate foods high in fat”, with 62.3% reporting poor diet; iii) item 4.1 – “monitored capillary blood glucose”, with 63.8% of unexpected responses; iv) item 4.2 – “monitored capillary blood glucose as recommended”, with 72.9% of unexpected responses; and v) item 5.1 – “examined feet”, with 64.3% reporting that they did not routinely assess foot health, considering the risk of developing lesions related to type 2 diabetes.

Activity 1.1, even with a larger number of people following a healthy diet, showed minimal difference between the scores of the expected response (50.8%) and the unexpected response (49.2%). The item with the best adherence was 2.3, related to the consumption of sweetened food. The other activities that presented scores within the expected range are those related to items 2.1, 5.2, 5.3, 6.1, 6.2 and 6.3. The frequencies related to the first six dimensions of the DSCQ are shown in Table 3.

**Table 3:** Absolute and relative frequency of adherence to activities for self-care of the first six dimensions of the Diabetes Self-Care Questionnaire (DSCQ). Goiânia, Goiás, 2024 (n=199)

DSCQ questions	Self-care frequency	
	≤4 days n (%)	>4 days n (%)
**1. Overall diet**		
1.1 Followed a healthy diet	98 (49.2)	101 (50.8)
1.2 Followed professional dietary guidance	127 (63.8)	72 (36.2)
**2. Specific diet**		
2.1 Ate fruit and/or vegetables	66 (33.2)	133 (66.8)
2.2 Ate food rich in fat	75 (37.7)	124 (62.3)
2.3 Ate sweetened food	188 (94.5)	11 (5.5)
**3. Physical activity**		
3.1 Did at least 30 minutes of physical activity	177 (88.9)	22 (11.1)
3.2 Did some sort of specific physical exercise	177 (88.9)	22 (11.1)
**4. Blood glucose monitoring**		
4.1 Tested capillary blood glucose	127 (63.8)	72 (36.2)
4.2 Tested capillary blood glucose as recommended	145 (72.9)	54 (27.1)
**5. Care of feet**		
5.1 Examined their feet	128 (64.3)	71 (35.7)
5.2 Examined the inside of their shoes before putting them on	62 (31.2)	137 (68.8)
5.3 Dried the spaces between the toes of their feet after washing them	72 (36.2)	127 (63.8)
6. Medication		
6.1 Took all medication as recommended	3 (3.5)	82 (96.5)
6.2 Administered insulin injections	1 (8.3)	11 (91.7)
6.3 Took the indicated number of tablets	4 (3.9)	98 (96.1)

The adherence scores for the “overall diet”, “physical activity” and “blood glucose monitoring” dimensions were 3.42 (3), 1.12 (1.98) and 2.58 (3), respectively. Adherence was observed to be below the expected level for self-care in relation to these dimensions, as they presented means lower than four.

When using multiple linear regression models, it was found that BMI showed a statistically significant result in the “overall diet” dimension, whereby higher BMI is associated with a poorer score (β=-0.21; 95%CI -0.38; -0.39; p-value<0.001). This dimension also showed statistical association with sex, whereby females demonstrated a more adequate diet (β=-0.15; 95%CI -0.30; -0.08; p-value 0.03). No statistically significant associations were found between the other variables [Table 4].

**Table 4:** Multiple linear regression models presented using the beta coefficient (β) and 95% confidence intervals (95%CI), taking the means of the Diabetes Self-Care Questionnaire (DSCQ) dimensions according to sociodemographic and clinical variables as continuous outcomes. Goiânia, Goiás, 2024 (n=199)

		Overall diet			Physical activity					Blood glucose monitoring		
Variables		β	95%CI	p-value		β	95%CI	p-value	β		95%CI	p-value
Alcoholism	-0.104		-0.269; 0.061	0.216		0.031	-0.077; 0.139	0.570	-0.197		-0.192; 0.118	0.639
Schooling	0.087		-0.055; 0.230	0.229		0.037	-0.056; 0.130	0.435	0.078		-0.056; 0.213	0.250
Age	0.044		-0.109; -0.197	0.570		-0.065	-0.165; 0.034	0.198	0.026		-0.118; 0.170	0.725
BMI^a^	-0.214		-0.388; -0.039	0.017		-0.020	-0.133; 0.094	0.730	0.053		-0.111; 0.217	0.523
Income	0.023		-0.229; 0.275	0.855		-0.140	-0.304; 0.025	0.095	-0.079		-0.316; 0.158	0.512
Time since diagnosis	0.013		-0.134; 0.159	0.866		0.033	-0.063; 0.128	0.502	0.067		-0.071; 0.205	0.338
Sex	-0.154		-0.300; -0.008	0.038		-0.082	-0.177; 0.013	0.090	0.088		-0.049; 0.225	0.208

^a^Body Mass Index.

Regarding the smoking dimension, 6.5% of participants had smoked at least one cigarette or taken at least one puff in the week prior to the interview. It was also observed that 66.8% had never smoked, 26.1% had not smoked for more than two years, 1.5% quit smoking between one and two years ago, 0.5% smoked their last cigarette in the month prior to the approach, and 5.1% had smoked on the day of the interview. The reported mean number of cigarettes consumed per day was 10 (5.55) units.

## Discussion

When assessing clinical profile and adherence to self-care in people with type 2 diabetes, a predominantly female sample was identified, mostly over 60 years of age and mostly classified as overweight or obese. The sample presented low adherence to self-care, inadequate lifestyle habits and clinical repercussions of the disease.

The predominance of females and individuals in their sixth decade of life can be justified by the large hormonal and metabolic changes typical in women at this stage, which increase the risks for type 2 diabetes [20]. The high frequency of overweight and obesity and the presence of inadequate dietary patterns, evidenced by the “overall diet” dimension of the DSCQ, point to the need for specific nutritional interventions, since excess weight worsens glycemic dysregulation and favors the progression of type 2 diabetes complications [21,22].

Regarding self-management of type 2 diabetes, assessed by the DSCQ, low adherence was observed among the participants and this behavior is influenced by several factors [23]. Individuals with low education levels are more likely to adopt negative attitudes towards self-management of the disease [24]. Although no statistically significant results regarding education were found in this sample, it was observed that more than 40% of individuals had low education levels, which may compromise their understanding of the disease, since it is a fundamental element for the development of the skills necessary for self-care management.

Time since diagnosis can also influence low adherence to diabetes self-management, since, as complications arise, motivation for self-care tends to decrease [25]. In this study, despite the absence of statistically significant relationships, most individuals reported more than 10 years since diagnosis, and most did not perform self-examination of the feet, this being a fundamental practice for preventing diabetic foot [26]. Although some preventive measures, such as checking footwear and drying the spaces between the toes, were mentioned, worrying rates of complications were found in this sample: 32.2% reported previous lesions, 11.0% had active lesions and 8.5% had already undergone amputations.

Low adherence to physical exercise was found. Most participants stated that they were sedentary. The “physical activity” dimension, as per items 3.1 and 3.2 of the DSCQ, showed the poorest performance among all dimensions assessed, with 88.9% reporting absence of physical activity in their daily routines and lack of regular exercise. These findings highlight the need to improve adherence to non-pharmacological interventions, because, despite their influence on glycemic control and their promoting discipline and autonomy in the self-care process, adherence to such practices is a challenge in this population [27].

Poor adherence to treatment can lead to chronic type 2 diabetes complications, triggering poor prognosis and metabolic disorders, potentially interfering with glycemic control [28]. Most participants in this study had complications related to type 2 diabetes. Among these, neuropathy was the most prevalent, followed by retinopathy and nephropathy. It is noteworthy that 21% of individuals reported the concomitant presence of two complications and 5% reported three complications, which makes the clinical picture more complex and potentially hinders therapeutic management [29].

Although people with type 2 diabetes generally have high rates of smoking, high sugar consumption and low consumption of fruit and vegetables [21], in this study, participants showed low prevalence of smoking, adequate consumption of fruit and/or vegetables and reduced sugar intake. This result can be attributed to specialized professional support in the treatment of type 2 diabetes, which provides guidance on nutrition, self-care and harm associated with inadequate habits. The good adherence observed in all items of the “medication” dimension also emphasizes the importance of professional support and guidance [30].

This study contributes to the understanding of the clinical profile and self-care practices in a specific population, highlighting the presence of important risk factors and barriers to diabetes self-management, assessed through a validated and specific questionnaire administered by experienced professionals. These findings highlight the need for the development of integrated approaches to improve care for these individuals. However, the study has limitations, including: the single-center design, convenience sampling and absence of follow-up laboratory tests. Further studies are recommended in order to assess the clinical repercussions of lack of self-management on the health of individuals with type 2 diabetes, as well as multicenter studies to broaden the assessment of self-care and the clinical profile of these patients.

The individuals with type 2 diabetes assessed by this study were predominantly women in their sixth decade of life, overweight and sedentary, who had been diagnosed for more than 10 years and at who had least one type 2 diabetes complication. Adherence to self-care, according to the DSCQ, was unsatisfactory, especially regarding diet, physical exercise, blood glucose monitoring and foot health assessment. Regression analysis indicated that individuals with higher BMI had poorer dietary habits, while females demonstrated more appropriate dietary choices.

## Data Availability

The database and the analysis codes used in this research are available upon request made by the peer reviewers.
