# Supplementary material for: Clinical profile and analysis of adherence to self-care in patients with type 2 diabetes: a cross-sectional study, Goiânia, Goiás, Brazil, 2024
Source: Epidemiol Serv Saude. 2026 May 2;35:e20250161. doi: 10.1590/S2237-96222026v35e20250161.en (PMC13686429; doi:10.1590/S2237-96222026v35e20250161.en)
Supplement: Translation

# Translation: Perfil clínico e análise da adesão ao autocuidado de pacientes com diabetes tipo 2: estudo transversal, Goiânia, Goiás, 2024

**DOI:** 10.1590/S2237-96222026v35e20250161.pt

**Authors:** Isabela Alves Cunha, Ana Caroline de Oliveira Lima, Lucas Raphael Bento Silva, Jordana Campos Martins de Oliveira, Geovana Cristina Batista Pacheco

**Affiliations:** 1 Hospital Estadual Alberto Rassi Comissão de Residência Multiprofissional Goiânia GO Brasil Hospital Estadual Alberto Rassi, Comissão de Residência Multiprofissional, Goiânia, GO, Brasil; 2 Centro Universitário Araguaia Goiânia GO Brasil Centro Universitário Araguaia, Goiânia, GO, Brasil

**Keywords:** Complicações do Diabetes, Autogestão, Endocrinologia, Doenças não Transmissíveis, Estudos Transversais

## Abstract

**Objetivo:**

Caracterizar o perfil clínico de pacientes com diabetes tipo 2 e analisar a adesão ao autocuidado desses indivíduos.

**Métodos:**

Estudo epidemiológico de corte transversal, realizado em um centro estadual especializado no atendimento do diabetes em Goiânia, Goiás, entre os meses de agosto e novembro de 2024. Foi realizada aplicação de questionário sociodemográfico, investigação do histórico de saúde, avaliação antropométrica e aplicação do Questionário de Atividades de Autocuidado com o Diabetes (QAD). Dados apresentados em frequência absoluta e relativa, média e desvio padrão. As associações foram analisadas por modelos de regressão linear múltipla, com intervalo de confiança de 95% (IC95%) e nível de significância de 5%.

**Resultados:**

A amostra de 199 participantes apresentou idade média de 64,9 (9,3) anos, predominância do sexo feminino (66,30%), mais de 10 anos de diagnóstico (67,30%) e mais de 80% com sobrepeso ou obesidade. Maioria de sedentários (84,40%), não fumantes (94,50%) e não etilistas (77,90%). Sobre complicações do diabetes, 45,60% relataram possuírem apenas uma, e a neuropatia foi a mais citada (64,32%), seguida de retinopatia (25,12%) e nefropatia (13,56%). O QAD revelou baixa adesão ao autogerenciamento, especialmente quanto à atividade física (88,90%), seguir orientações alimentares profissionais (63,80%), evitar alimentos gordurosos (62,30%), monitorizar a glicemia (63,80%) e examinar os pés (64,30%). A maioria dos itens do QAD apresentou adesão insatisfatória.

**Conclusão:**

Tratam-se de indivíduos na sexta década de vida, com sobrepeso ou obesidade, com mais de 10 anos de diagnóstico e com baixa adesão ao autocuidado.

**Aspectos éticos**

Esta pesquisa respeitou os princípios éticos, obtendo os seguintes dados de aprovação:

Comitê de ética em pesquisa: Hospital Estadual Alberto Rassi

Número do parecer: 6.030.934

Data de aprovação: 17/4/2023

Certificado de apresentação de apreciação ética: 67329423.4.0000.0035

Registro do consentimento livre e esclarecido: Obtido de todos os participantes antes da coleta.

## Introdução

O diabetes é uma patologia crônica e metabólica caracterizada pelo acúmulo de glicose sérica, influenciada por fatores externos e condições clínicas [1]. Tem etiologia composta por fatores de risco não modificáveis, como idade, sexo e hereditariedade, e mutáveis, como os hábitos de vida [2]. O diabetes classifica-se em dois tipos: o tipo 1, que apresenta forte predisposição genética e exige tratamento contínuo com insulina [3]; e o tipo 2, que, embora também possua componente genético, seu controle está relacionado a mudanças no estilo de vida [4].

A ocorrência do diabetes tipo 2 cresce com o avanço da idade e apresenta maior incidência entre 55 e 59 anos, acometendo os homens em faixas etárias mais precoces [5]. Em 2021, o Brasil foi o sexto país com maior número de pessoas com a doença e, de acordo com estimativas, esse número deve aumentar 47% em 20 anos [6]. 

A rotina de autocuidado no diabetes tipo 2 deve incluir o gerenciamento de medicações e hábitos de vida, como alimentação adequada, prática de exercício físico, ingestão de água, interrupção de etilismo e/ou tabagismo e vigilância da integridade dos pés para prevenir complicações crônicas da doença [7]. Entretanto, esses indivíduos geralmente possuem hábitos inadequados e menor adesão a tratamentos não farmacológicos [8]. O mau gerenciamento do diabetes tipo 2 influencia no aparecimento de novas condições [9] e os indivíduos podem apresentar, por exemplo, índice de massa corporal (IMC) elevado, deposição lipídica e desajustes osteomioarticulares [8]. 

Nesse contexto, instrumentos de avaliação do autocuidado são importantes para mapear os principais fatores prejudiciais à saúde e qualidade de vida de pessoas com diabetes tipo 2 [10]. A utilização de questionários que analisam o nível de adesão ao tratamento desses pacientes permite a identificação de problemas e a necessidade de direcionamento para um plano de autocuidado associado ao manejo clínico [10]. Para tal, o Questionário de Atividades de Autocuidado com o Diabetes (QAD) é um instrumento de alta confiabilidade, que avalia o nível de adesão ao autocuidado pela análise de frequência da prática de determinadas atividades semanalmente [11]. 

Diante disso, torna-se necessário investigar a adesão dos indivíduos com diabetes tipo 2 quanto às suas atividades de manejo para identificar os aspectos que influenciam essa prática. Este estudo teve o objetivo de caracterizar o perfil clínico de pacientes com diabetes tipo 2 e analisar a adesão ao autocuidado desses indivíduos.

## Metodologia

### Delineamento do estudo

Estudo epidemiológico de corte transversal.

### Contexto

Este estudo foi conduzido em um centro estadual de referência no atendimento a pessoas com diabetes, localizado em Goiânia, capital do estado de Goiás, no período de agosto a novembro de 2024. Dados da Secretaria Estadual de Saúde indicam que, em Goiás, a prevalência autorreferida de diabetes entre adultos é de 6,4% [12]. Além da prevalência, a taxa de internações hospitalares atribuídas ao diabetes alcançou 57,44%, com os maiores custos associados à idosos com complicações crônicas da doença [13]. Tais complicações, frequentemente decorrentes da ausência ou inadequação do autocuidado, constituem o principal foco desta investigação, dado seu impacto significativo sobre a morbimortalidade e os custos em saúde [8,9].

### Participantes

Foram incluídos no estudo indivíduos com idade superior a 18 anos, de ambos os sexos, diagnosticados com diabetes tipo 2, com tempo mínimo de três meses de acompanhamento na instituição de saúde, e que assinaram o Termo de Consentimento Livre e Esclarecido (TCLE). Os critérios de exclusão foram indivíduos incapazes de responder à anamnese por questões cognitivas e/ou biológicas ou que optaram por retirar seu consentimento de participação da pesquisa.

Os pesquisadores apresentaram o TCLE e informaram os voluntários sobre os objetivos e procedimentos aos quais seriam submetidos, assim como todos os riscos e benefícios.

### Variáveis

As variáveis dependentes foram os comportamentos de autocuidado medidos pelo QAD, contemplados pelos domínios “alimentação geral”, “alimentação específica”, “atividade física”, “monitorização da glicemia”, “cuidado com os pés”, “uso de medicação” e “tabagismo”.

As variáveis independentes foram:

Dados sociodemográficos:

- sexo (masculino; feminino);
- faixa etária (em anos: 18 a 29; 30 a 39; 40 a 49; 50 a 59; 60 a 69; 70 a 79; 80 a 85);
- renda mensal (abaixo de um salário-mínimo; de um a dois salários-mínimos; maior que dois salários-mínimos); e
- escolaridade (sem escolaridade; fundamental incompleto; fundamental completo; médio incompleto; médio completo; superior incompleto; superior completo).

Dados clínicos:

- peso (em quilogramas – kg);
- altura (em metros – m);
- IMC (em quilogramas por metro quadrado – kg/m^2^; por classificação: eutrófico, sobrepeso, obesidade);
- tabagismo (sim; não);
- etilismo (sim; não);
- tempo de diagnóstico (<10 anos de diagnóstico; ≥10 anos de diagnóstico);
- prática de exercício físico (ativo; sedentário);
- terapia medicamentosa (comprimidos; insulinoterapia; ambos);
- número de complicações (nenhuma; apenas uma; duas associadas; três associadas);
- amputação (sim; não);
- histórico de lesões (sim; não); e
- lesões agudas (sim; não).

### Fontes de dados e mensuração

Os dados foram coletados por entrevistas e aplicação de questionários por profissionais treinados e com experiência no tratamento do diabetes tipo 2. A linguagem foi adaptada ao público para garantir compreensão dos assuntos abordados e fidedignidade das respostas. Os déficits cognitivos foram determinados com base na percepção dos profissionais durante a entrevista, complementada pela análise de registros em prontuário clínico.

Para coleta de dados sociodemográficos e histórico de saúde foi utilizado um questionário sociodemográfico, construído pelos próprios autores, com perguntas referentes a idade, sexo, renda mensal, nível de escolaridade, peso, altura, IMC, tempo de diagnóstico de diabetes tipo 2, complicações diabéticas, terapia medicamentosa, tabagismo e etilismo, prática de exercícios físicos, histórico de feridas nos pés e de amputação. 

Para avaliação do autocuidado foi aplicado o QAD, que é um questionário revisado e padronizado com medidas de score e que avalia a adesão de indivíduos com diabetes tipo 2 ao autocuidado no tratamento da doença [14]. Foi utilizada a versão traduzida, adaptada e validada para a língua portuguesa [15]. O QAD é composto por 15 itens que analisam cinco aspectos do tratamento do diabetes, agrupados em sete dimensões do autocuidado: i) alimentação geral; ii) alimentação específica; iii) atividade física; iv) monitorização da glicemia; v) cuidado com os pés; vi) uso de medicação; e vii) tabagismo [16]. 

Para cada item, o paciente é questionado sobre a quantidade de dias por semana que pratica determinada atividade de autocuidado [16]. As alternativas variam de 0 a 7 dias e considera-se “0 dias” a resposta menos esperada, indicando menor adesão ao autocuidado, e “7 dias” a mais esperada, indicando melhor autogerenciamento da doença [17]. Porém, na dimensão “alimentação específica”, nos dois últimos itens, os valores são invertidos, pois questiona-se quanto ao consumo de alimentos ricos em gordura e açúcar [17]. Para estes itens, a resposta “0 dias” é a mais esperada, e a resposta “7 dias” é a menos esperada [17].

Atribui-se a classificação de comportamento desejável quando a média de dias para cada item é superior a 4 ou, no caso dos itens 2.2 e 2.3 da dimensão “alimentação específica”, quando a média de dias é ≤4 [18]. As dimensões “alimentação geral”, “atividade física” e “monitorização da glicemia”, por possuírem boas correlações entre seus itens, foram avaliadas por média total de dias entre eles [15]. Já os itens das outras dimensões foram avaliados individualmente e apresentados em frequência, devido à fraca correlação entre eles [15].

### Viés

Alguns dados foram baseados em informações autorreferidas, sujeitas a viés de informação e viés de recordatório, em que os participantes poderiam esquecer de aspectos relevantes quanto ao seu histórico de saúde. 

### Tamanho do estudo

Trata-se de amostra não probabilística por conveniência. A amostra foi baseada na agenda dos profissionais de um centro estadual especializado, incluindo médicos, nutricionistas, podólogos, enfermeiros e fisioterapeutas. Os pacientes convidados a participar da pesquisa foram abordados às quartas e sextas-feiras no período matutino, enquanto aguardavam suas consultas na sala de espera. Após a abordagem, eram encaminhados para uma sala reservada, com boa iluminação e climatização, para garantir a privacidade dos pacientes.

Considerando uma média de 15 pacientes por período, utilizou-se um tempo de quatro meses para a coleta de dados, de agosto a novembro de 2024. Essa estratégia teve como objetivo alinhar a seleção à rotina da instituição. Os estágios de seleção estão representados na Figura 1.

**Figura 1:** Etapas para elegibilidade e seleção dos participantes do estudo. Goiânia, Goiás, 2024 (n=199)

### Métodos estatísticos

Em termos descritivos, as variáveis contínuas foram expressas por média e desvio padrão, e as variáveis categóricas foram apresentadas em frequência absoluta e relativa.

Para investigar associações entre variáveis sociodemográficas, clínicas e os scores de autocuidado (avaliados por meio do QAD), foram utilizados modelos de regressão linear múltipla, considerando como desfechos contínuos as médias das dimensões do QAD. O modelo foi ajustado por variáveis como sexo, idade, IMC, tempo de diagnóstico, escolaridade e renda. Os pressupostos dos modelos de regressão linear foram checados, incluindo normalidade dos resíduos, homocedasticidade e ausência de multicolinearidade. Os resultados foram expressos pelos coeficientes beta (β) estimados, com respectivos intervalos de confiança de 95% (IC95%). Os dados foram analisados utilizando o software Statistical Package for the Social Sciences (SPSS), versão 25.0 e adotou-se um nível de significância fixo de 5%.

A significância estatística foi interpretada com cautela, considerando também a magnitude dos efeitos e sua relevância clínica, conforme recomendações metodológicas atuais na área da saúde coletiva [19].

## Resultados

O estudo foi realizado com amostra de 199 pacientes. A idade média foi de 64,9 (9,3) anos, sendo 43,7% pertencentes à sexta década de vida e com predominância do sexo feminino (66,3%). A maior parte dos indivíduos relatou arrecadar mais de um salário-mínimo por mês (92,0%). Apenas 1,5% dos indivíduos alcançaram a graduação e 12,1% nunca frequentaram instituições de ensino [Tabela 1]. 

**Tabela 1:** Caracterização sociodemográfica da amostra apresentada pela frequência absoluta e relativa. Goiânia, Goiás, 2024 (n=199)

Características da amostra	Valores n (%)
Sexo	
Masculino	67 (33,7)
Feminino	132 (66,3)
**Faixa etária **(anos)	
18-29	0 (0)
30-39	2 (1)
40-49	9 (4,5)
50-59	43 (21,6)
60-69	87 (43,7)
70-79	48 (24,1)
80-85	10 (5,1)
**Renda mensal **(salários-mínimos)	
<1	16 (8)
1-2	118 (59,3)
>2	65 (32,7)
Escolaridade	
Sem escolaridade	24 (12,1)
Fundamental incompleto	92 (46,2)
Fundamental completo	12 (6)
Médio incompleto	16 (8)
Médio completo	49 (24,6)
Superior incompleto	3 (1,5)
Superior completo	3 (1,5)

A amostra apresentou peso médio de 77,1 (16,1) kg e altura de 1,61 (0,09) m, perfazendo IMC médio de 29,6 (5,7) kg/m^2^, com 41,2% classificados com sobrepeso e 40,2% com obesidade. A maioria da amostra foi composta por não fumantes (94,5%), não etilistas (77,9%) e sedentários (84,4%). A maioria possuía mais de 10 anos de diagnóstico (67,3%), utilizava unicamente comprimidos como terapia medicamentosa (51,3%) e apresentava apenas uma complicação relacionada ao diabetes tipo 2 (45,6%). Ainda sobre as complicações, a neuropatia foi a mais citada (64,32%), seguida da retinopatia (25,12%) e da nefropatia (13,56%). Demais dados de caracterização do perfil clínico da amostra estão apresentados na Tabela 2.

**Tabela 2:** Caracterização do perfil clínico de pacientes com diabetes tipo 2 apresentada pela frequência absoluta e relativa. Goiânia, Goiás, 2024 (n=199)

Variável	Valores n (%)
**Classificação IMC^a^**	
Eutrófico	37 (18,6)
Sobrepeso	82 (41,2)
Obesidade	80 (40,2)
**Tabagismo **	
Fumante	11 (5,5)
Não fumante	188 (94,5)
**Etilismo **	
Etilista	44 (22,1)
Não etilista	155 (77,9)
**Tempo de diagnóstico **(anos)	
<10	65 (32,7)
≥10	134 (67,3)
**Prática de exercício físico**	
Ativo	31 (15,6)
Sedentário	168 (84,4)
**Terapia medicamentosa**	
Comprimidos	102 (51,3)
Insulinoterapia	12 (6)
Ambos	85 (42,7)
**Internação nos últimos dois anos por descompensação do diabetes**	
Sim	33 (16,6)
Não	166 (83,4)
**Número de complicações do diabetes**	
Nenhuma	56 (28,4)
Apenas uma	91 (45,6)
Duas associadas	42 (21)
Três associadas	10 (5)
**Presença de amputação**	
Possui amputação	17 (8,5)
Não possui amputação	182 (91,5)
**Histórico de lesões nos pés **	
Sim	64 (32,2)
Não	135 (67,8)
**Presença atual de feridas nos pés**	
Sim	21 (11)
Não	178 (89)

^a^Índice de Massa Corporal.

As atividades do QAD sobre prática de exercício físico (itens 3.1 e 3.2) foram as que apresentaram pior adesão. Outras atividades que apresentaram baixa adesão foram: i) item 1.2 – “seguiu uma orientação alimentar profissional”, com 63,8% de relatos com frequência menor que 4 dias por semana; ii) item 2.2 – “comeu alimentos ricos em gordura”, com 62,3% de relatos de má alimentação; iii) item 4.1 – “avaliou a glicemia capilar”, com 63,8% de respostas não esperadas; iv) item 4.2 – “avaliou a glicemia capilar conforme recomendado”, com 72,9% de respostas também não esperadas; e v) item 5.1 – “examinou os pés”, com relato de 64,3% das pessoas que não mantinham o costume de avaliar a saúde dos pés, uma vez que deve ser considerado o risco de desenvolverem lesões relacionadas ao diabetes tipo 2.

A atividade do item 1.1, mesmo com um número maior de pessoas que seguiram uma dieta saudável, apresentou diferença mínima entre os scores da resposta esperada (50,8%) e da resposta não esperada (49,2%). O item que apresentou melhor adesão foi o 2.3, referente ao consumo de doces. As demais atividades que apresentaram score dentro do esperado são as relacionadas aos itens 2.1, 5.2, 5.3, 6.1, 6.2 e 6.3. As frequências relacionadas às seis primeiras dimensões do QAD estão apresentadas na Tabela 3.

**Tabela 3:** Frequência absoluta e relativa da adesão às atividades de autocuidado das seis primeiras dimensões do Questionário de Autocuidado com o Diabetes (QAD). Goiânia, Goiás, 2024 (n=199)

Questões QAD	Frequência do autocuidado	
	≤4 dias n (%)	>4 dias n (%)
**1. Alimentação geral**		
1.1 Seguiu uma dieta saudável	98 (49,2)	101 (50,8)
1.2 Seguiu uma orientação alimentar profissional	127 (63,8)	72 (36,2)
**2. Alimentação específica**		
2.1 Comeu frutas e/ou vegetais	66 (33,2)	133 (66,8)
2.2 Comeu alimentos ricos em gordura	75 (37,7)	124 (62,3)
2.3 Comeu doces	188 (94,5)	11 (5,5)
**3. Atividade física**		
3.1 Praticou pelo menos 30 minutos de atividade física	177 (88,9)	22 (11,1)
3.2 Praticou algum exercício físico específico	177 (88,9)	22 (11,1)
**4. Monitorização da glicemia**		
4.1 Avaliou a glicemia capilar	127 (63,8)	72 (36,2)
4.2 Avaliou a glicemia capilar conforme recomendado	145 (72,9)	54 (27,1)
**5. Cuidado com os pés**		
5.1 Examinou os pés	128 (64,3)	71 (35,7)
5.2 Examinou dentro dos sapatos antes de calçá-los	62 (31,2)	137 (68,8)
5.3 Secou os espaços entre os dedos dos pés depois de lavá-los	72 (36,2)	127 (63,8)
6. Medicação		
6.1 Tomou todos os medicamentos conforme recomendado	3 (3,5)	82 (96,5)
6.2 Administrou injeções de insulina	1 (8,3)	11 (91,7)
6.3 Tomou o número indicado de comprimidos	4 (3,9)	98 (96,1)

Os *scores *de aderência apresentados pelas dimensões “alimentação geral”, “atividade física” e “monitorização da glicemia” foram 3,42 (3), 1,12 (1,98) e 2,58 (3), respectivamente. Observou-se aderência abaixo do esperado para o autocuidado referente a essas dimensões, por apresentarem médias menores que quatro.

Por meio de modelos de regressão linear múltipla, verificou-se que o IMC apresentou resultado estatisticamente significativo na dimensão “alimentação geral”, em que maior IMC está associado a pior pontuação (β=-0,21; IC95% -0,38; -0,39; p-valor<0,001). Essa mesma dimensão também apresentou associação estatística ao sexo, em que as mulheres demonstraram alimentação mais adequada (β=-0,15; IC95% -0,30; -0,08; p-valor 0,03). Não foram encontradas associações estatisticamente significativas entre as demais variáveis [Tabela 4].

**Tabela 4:** Modelos de regressão linear múltipla apresentados por coeficiente beta (β) e intervalos de confiança de 95% (IC95%), considerando como desfechos contínuos as médias das dimensões do Questionário de Autocuidado com o Diabetes (QAD) pelas variáveis sociodemográficas e clínicas. Goiânia, Goiás, 2024 (n=199)

		Alimentação geral			Atividade física					Monitorização da glicemia		
Variáveis		β	IC95%	p-valor		β	IC95%	p-valor	β		IC95%	p-valor
Alcoolismo	-0,104		-0,269; 0,061	0,216		0,031	-0,077; 0,139	0,570	-0,197		-0,192; 0,118	0,639
Escolaridade	0,087		-0,055; 0,230	0,229		0,037	-0,056; 0,130	0,435	0,078		-0,056; 0,213	0,250
Idade	0,044		-0,109; -0,197	0,570		-0,065	-0,165; 0,034	0,198	0,026		-0,118; 0,170	0,725
IMC^a^	-0,214		-0,388; -0,039	0,017		-0,020	-0,133; 0,094	0,730	0,053		-0,111; 0,217	0,523
Renda	0,023		-0,229; 0,275	0,855		-0,140	-0,304; 0,025	0,095	-0,079		-0,316; 0,158	0,512
Tempo de diagnóstico	0,013		-0,134; 0,159	0,866		0,033	-0,063; 0,128	0,502	0,067		-0,071; 0,205	0,338
Sexo	-0,154		-0,300; -0,008	0,038		-0,082	-0,177; 0,013	0,090	0,088		-0,049; 0,225	0,208

^a^Índice de Massa Corporal.

Quanto à dimensão relativa ao tabagismo, 6,5% dos participantes fumaram no mínimo um cigarro ou deram no mínimo uma tragada na semana anterior à entrevista. Observou-se, também, que 66,8% nunca fumaram, 26,1% não fumam há mais de dois anos, 1,5% pararam de fumar entre um e dois anos atrás, 0,5% fumaram seu último cigarro no mês anterior à abordagem e 5,1% haviam fumado no dia da entrevista. A média relatada de cigarros consumidos por dia foi de 10 (5,55) unidades.

## Discussão

Ao avaliar o perfil clínico e a adesão ao autocuidado de pessoas com diabetes tipo 2, foi identificada uma amostra predominantemente do sexo feminino, com idade superior a 60 anos, em sua maioria classificados com sobrepeso e obesidade. A amostra apresentou baixa adesão ao autocuidado, hábitos de vida inadequados e repercussões clínicas da doença.

A predominância do sexo feminino e de indivíduos na sexta década de vida pode ser justificada pela grande alteração hormonal e metabólica típicas em mulheres nessa fase, que elevam os riscos para diabetes tipo 2 [20]. A elevada frequência de sobrepeso e obesidade e a presença de padrões alimentares inadequados, evidenciada pela dimensão “alimentação geral” do QAD, apontam para a necessidade de intervenções nutricionais específicas, uma vez que o excesso de peso agrava o descontrole glicêmico e favorece a progressão das complicações do diabetes tipo 2 [21,22].

Quanto ao autogerenciamento do diabetes tipo 2, avaliado pelo QAD, verificou-se baixa adesão entre os indivíduos, e esse comportamento é influenciado por diversos fatores [23]. Os indivíduos com baixa escolaridade apresentam maior propensão a adotar atitudes negativas em relação ao autogerenciamento da doença [24]. Embora não tenham sido encontrados nesta amostra resultados estatisticamente significativos referentes à escolaridade, foi observado que mais de 40% dos indivíduos apresentaram baixa escolaridade, o que pode comprometer a compreensão acerca da doença, visto que constitui elemento fundamental para o desenvolvimento das competências necessárias à gestão do autocuidado.

O tempo de diagnóstico também pode influenciar na baixa adesão ao autogerenciamento do diabetes, uma vez que, à medida que surgem complicações, a motivação para o autocuidado tende a diminuir [25]. Neste estudo, apesar da ausência de relações estatisticamente significativas, a maior parte dos indivíduos relatou mais de 10 anos de diagnóstico, e a maioria não realizava o autoexame dos pés, uma prática fundamental para prevenir o pé diabético [26]. Embora algumas medidas preventivas, como a verificação dos calçados e a secagem dos espaços interdigitais, tenham sido referidas, foram constatados, nesta amostra, índices preocupantes de complicações: 32,2% relataram lesões prévias, 11,0% apresentavam feridas ativas e 8,5% já haviam sido submetidos a amputações.

Observou-se baixa adesão à prática de exercício físico. A maior parte dos participantes declarou-se sedentária. A dimensão “atividade física”, nos itens 3.1 e 3.2 do QAD, apresentou o pior desempenho entre todas as dimensões avaliadas, com 88,9% relatando ausência de atividade física nas rotinas diárias e de prática regular de exercícios. Esses achados evidenciam a necessidade de melhorar a adesão às intervenções não farmacológicas, pois, apesar de sua influência no controle glicêmico e na promoção de disciplina e autonomia no processo de autocuidado, a adesão a tais práticas é um desafio nessa população [27].

A baixa adesão ao tratamento pode provocar complicações crônicas do diabetes tipo 2, que desencadeiam um cenário de mau prognóstico e desordem nos fatores metabólicos, podendo interferir no controle glicêmico [28]. A maior parte dos participantes deste estudo possuem complicações relacionadas ao diabetes tipo 2. Dentre essas, a neuropatia foi a mais prevalente, seguida da retinopatia e nefropatia. Ressalta-se que 21% dos indivíduos relataram a presença concomitante de duas complicações e 5% relataram três complicações, o que torna o quadro clínico mais complexo e que potencialmente dificulta o manejo terapêutico [29].

Embora pessoas com diabetes tipo 2 apresentem, em geral, altas taxas de tabagismo, elevado consumo de açúcares e baixo consumo de frutas e vegetais [21], neste estudo, os participantes apresentaram baixa prevalência de tabagismo, consumo adequado de frutas e/ou vegetais e reduzida ingestão de açúcares. Esse resultado pode ser atribuído ao suporte profissional especializado no tratamento do diabetes tipo 2, que realiza orientações sobre alimentação, autocuidado e os prejuízos associados a hábitos inadequados. A boa adesão observada em todos os itens da dimensão “medicação” reforça, igualmente, a importância do suporte e do direcionamento profissional [30].

Este estudo contribui para a compreensão do perfil clínico e das práticas de autocuidado em uma população específica, destacando a presença de importantes fatores de risco e barreiras ao autogerenciamento do diabetes, avaliada por meio de questionário validado e específico, sendo realizada por profissionais experientes. Essas descobertas evidenciam a necessidade do desenvolvimento de abordagens integradas para melhorar a atenção a esses indivíduos. Contudo, o estudo apresenta limitações, entre elas: o formato unicêntrico, a amostragem por conveniência e a ausência de acompanhamento de exames laboratoriais. Recomenda-se a realização de estudos que avaliem as repercussões clínicas da falta de autogerenciamento na saúde dos indivíduos com diabetes tipo 2 e estudos multicêntricos para ampliar a avaliação do autocuidado e do perfil clínico dos pacientes.

Os indivíduos com diabetes tipo 2 avaliados foram predominantemente mulheres na sexta década de vida, com sobrepeso e sedentários, com mais de 10 anos de diagnóstico e pelo menos uma complicação da doença. A adesão ao autocuidado, segundo o QAD, foi insatisfatória, especialmente em alimentação, exercício físico, monitorização da glicemia e avaliação da saúde dos pés. A regressão apontou que indivíduos com maior IMC apresentaram piores hábitos alimentares, enquanto as mulheres demonstraram escolhas alimentares mais adequadas.

## Data Availability

O banco de dados e os códigos de análise utilizados na pesquisa estão disponíveis sob demanda dos pareceristas.
